# Comparison of Oropharyngeal Microbiota in Healthy Piglets and Piglets With Respiratory Disease

**DOI:** 10.3389/fmicb.2018.03218

**Published:** 2018-12-21

**Authors:** Qun Wang, Rujian Cai, Anni Huang, Xiaoru Wang, Wan Qu, Lei Shi, Chunling Li, He Yan

**Affiliations:** ^1^School of Food Science and Engineering, South China University of Technology, Guangzhou, China; ^2^Institute of Animal Health, Guangdong Academy of Agricultural Sciences, Guangzhou, China; ^3^Institute of Food Safety and Nutrition, Jinan University, Guangzhou, China; ^4^State Key Laboratory of Food Safety Technology for Meat Products, Xiamen, China

**Keywords:** porcine respiratory disease, piglets, 16S rRNA sequencing, oropharyngeal microbiota, microbial community

## Abstract

Porcine respiratory disease (PRD) is responsible for severe economic losses in the swine industry worldwide. Our objective was to characterize the oropharyngeal microbiota of suckling piglets and compare the microbiota of healthy piglets and piglets with PRD. Oropharyngeal swabs were collected from healthy (Healthy_A, *n* = 6; Healthy_B, *n* = 4) and diseased (PRD_A, *n* = 18; PRD_B, *n* = 5) piglets at 2–3 weeks of age from two swine farms in Guangdong province, China. Total DNA was extracted from each sample and the V3-V4 hypervariable region of the 16S rRNA gene was amplified and sequenced using the Illumina MiSeq platform. No statistically significant differences were observed in bacterial diversity and richness between the healthy and PRD groups in the two farms except for Shannon index in farm A. Principal coordinates analysis (PCoA) showed structural segregation between diseased and healthy groups and between groups of different farms. Among all samples, the phyla *Firmicutes*, *Proteobacteria*, and *Bacteroidetes* were predominant. At the genus level, *Streptococcus*, *Lactobacillus*, and *Actinobacillus* were the core genera in the oropharynx of healthy piglets from the two farms. Significant differences in bacterial taxa were found when the microbiota was compared regarding the health status. In farm A, the percentages of *Moraxella* and *Veillonella* were higher in the PRD group, while only *Porphyromonas* was significantly increased in the PRD group in farm B (*p* < 0.05). Compared to PRD groups, statistically significant predominance of *Lactobacillus* was observed in the healthy groups from both farms (*p* < 0.05). Our findings revealed that *Moraxella*, *Veillonella*, and *Porphyromonas* may play a potential role in PRD and *Lactobacillus* may have a protective role against respiratory diseases.

## Introduction

Porcine respiratory disease (PRD) is a major challenge in swine farms and can result in significant economic losses because of increased mortality and morbidity, escalated treatment costs, reduced growth rates, and low feed conversion efficiency (Holt et al., 2011). It is a multifactorial disease caused by a combination of infectious viral or bacterial pathogens as well as adverse environmental conditions and presents with clinical symptoms, including cough, dyspnoea, fever, and anorexia (Zhao et al., 2011; Chae, 2016). Several bacterial and viral agents are involved in the pathogenesis of this disease, including swine influenza virus (SIV), porcine reproductive and respiratory syndrome virus (PRRSV), *Actinobacillus pleuropneumoniae*, *Pasteurella multocida*, and *Mycoplasma hyopneumoniae* (Pomorska-Mól et al., 2017). Respiratory pathogens can be divided into primary pathogens, which can induce severe lesions in respiratory tissues, and secondary or opportunistic infectious pathogens that need support from other co-infecting pathogens or cofactors to induce substantial lesions in the respiratory system (Opriessnig et al., 2011).

The oropharynx serves the respiratory and digestive systems and is colonized by bacterial pathogens that affect healthy or immunocompromised individuals (Lu et al., 2015). The microbial composition of the respiratory tract is increasingly considered as an important source of biomarkers for non-invasive diagnosis of diseases (Lu et al., 2015). A better understanding of the significance of normal microbial community structure, and its variations in disease states could provide critical insights into the pathogenesis of respiratory diseases, as well as provide a new prevention strategy to control PRD. Culture-dependent techniques are often used to determine the bacterial populations, however, most microbes are difficult to be cultured in the laboratory (Sharpton, 2014). With the development of next-generation sequencing, it is possible to investigate the microbiome (Lowe et al., 2012; Knudsen et al., 2016; Burrough et al., 2017).

Most porcine microbiota studies have focused on the intestinal tract, while the microbial populations related to upper respiratory tract are poorly understood. The tonsils and nose have been used to explore the upper respiratory microbial populations (Lowe et al., 2011; Weese et al., 2014). However, there has been limited investigation on the oropharyngeal microbiota, especially on those associated with respiratory diseases. Our goals were to identify the core bacterial genera in the oropharynx of healthy piglets and compare the oropharyngeal bacterial community in healthy piglets with those affected with PRD.

## Materials and Methods

### Ethics Statement

This study was carried out in accordance with the recommendations of the experimental animal administration and ethics committee of South China University of Technology of guidelines. The protocol was approved by the Institutional Animal Care and Use Committee of South China University of Technology.

### Sample Collection

This study was conducted at two commercial swine farms, each with more than 10,000 pigs. The two farms located at least 100 km apart in Guangdong province, Southern China. Both farms practiced intensive, farrow-to-finish, open-house systems. The piglets from the same farm were raised under the same standard indoor conditions according to their growth stages. In detail, the piglets were raised in farrowing house from birth to 28 days old. Then, the piglets were moved to nursery pens until their weight reached approximately 30 kg. Subsequently, the animals were moved to growing–finishing pens to be sold out as breeders. Dominant breeds in the two farms were crosses between Landrace and Yorkshire (sows) with Duroc or Hampshire (boars). Sample collection was carried out in March 2017. Oropharyngeal swabs were taken from 33 piglets at 2–3 weeks of age before weaning from the two swine farms. Twenty four oropharyngeal samples (18 PRD and 6 healthy) were collected from farm A and 9 oropharyngeal samples (5 PRD and 4 healthy) were collected from farm B. Veterinarians with over 5 years of experience diagnosed PRD by physical examination. The piglets with PRD presented with clinical symptoms, including cough, fever, joint swelling, and wheezes. Only those piglets that had never experienced PRD and other diseases were included in the healthy groups. Deep oropharyngeal swabs were collected from the piglets with the help of the veterinarians using 20 cm DNA-free sterile swabs covered with a sterile plastic sheath. All samples were kept on ice until they were transferred to the laboratory where they were resuspended in 500 μL PBS and stored at -80°C until processing.

### DNA Extraction and PCR Amplification

DNA was extracted from the oropharyngeal swabs from the piglets using the QIAamp Mini Kit (Qiagen, Hilden, Germany) following the manufacturer’s instructions. The final DNA concentration was determined on the basis of the absorbance at 260 and 280 nm using a Nanodrop 2000 UV-vis spectrophotometer (Thermo Fisher Scientific, Wilmington, United States), and the DNA quality was evaluated by 1% agarose gel electrophoresis. Negative controls were performed for the extraction process and evaluated by gel analysis after PCR amplification. For metagenomic analysis, the V3-V4 region of the 16S rRNA gene was amplified using forward (338F, 5′ACTCCTACGGGAGGCAGCAG-3′) and reverse (806R, 5′-GGACTACHVGGGTWTCTAAT-3′) primers on a GeneAmp^®^ 9700 (ABI, United States) PCR system. PCR reactions were performed in triplicate 20 μL mixture containing 10 ng template DNA, 4 μL 5 × FastPfu buffer, 2 μL dNTPs (2.5 mmol/L), 0.8 μL each primer (5.0 μmol/L), 0.4 μL FastPfu polymerase and ddH_2_O. The reaction conditions for PCR were 95°C for 3 min for an initial denaturation, followed by 27 cycles of denaturation at 95°C for 30 s, primer annealing at 55°C for 30 s, extension at 72°C for 45 s, and a final elongation for 10 min at 72°C. The PCR products were extracted from 2% agarose gels and further purified using the AxyPrep DNA Gel Extraction Kit (Axygen Biosciences, Union City, CA, United States) and quantified using QuantiFluor^TM^ -ST (Promega BioSciences LLC, Sunnyvale, CA, United States) according to the manufacturer’s protocol. Samples that contained no template and those that contained known 16S rRNA gene sequences were used as negative and positive controls in the PCR reactions. DNA was stored at -80°C prior to downstream processing.

### Illumina MiSeq Sequencing

The DNA samples were quantified by spectrophotometry (Thermo Fisher Scientific, Wilmington, United States) and diluted to a final concentration of 2 nM. Purified amplicons were sequenced by paired-end sequencing (2 × 300) on an Illumina MiSeq platform (San Diego, CA, United States) for metagenomic analysis.

### Bioinformatics Analysis

All raw reads were demultiplexed and quality-filtered by Trimmomatic and merged by FLASH with the following criteria: (1) The reads were truncated at any site receiving an average quality score less than 20 over a 50 bp sliding window. (2) Primers were exactly matched allowing two nucleotides mismatching and reads containing ambiguous bases were discarded from the analysis. (3) Sequences whose overlap was longer than 10 bp were merged according to their overlap sequence.

Operational taxonomic units (OTUs) were clustered with 97% similarity cutoff using UPARSE (version 7.1^1^) and chimeric sequences were identified and removed using the UCHIME tool. RDP Classifier^2^ was used to analyze the phylogenetic affiliation of each 16S rRNA gene sequence, against the silva (SSU115) 16S rRNA database with a confidence threshold of 70%. Sequences belonging to non-bacterial domains, including chloroplasts, mitochondria, archaea, and eukaryotes, were removed.

### Statistical Analysis

The analysis was performed at different taxonomic levels: kingdom, phylum, class, order, family, genus, and species. OTUs that reached 97% similarity were used for alpha diversity estimations, which included observed OTUs (Sobs), diversity (Shannon and Simpson indices), richness (Chao I), coverage (Good’s coverage), and rarefaction curve analysis using Mothur (Version 1.30.2 ^3^). Alpha diversity metrics between different groups were compared using Student’s *t*-test and *p* < 0.05 was considered a statistically significant difference. The core microbiota (OTUs with >1% relative abundance and shared in all healthy piglets) was explored for the healthy piglets in the two farms. Bray–Curtis distance metrics analysis was performed using OTUs for each sample, and principal coordinates analysis (PCoA) analysis was conducted according to this distance. Venn diagram analysis was performed to show unique and shared OTUs in the microbial population structure between healthy and diseased groups. Heatmaps were generated with the R-package gplots at the phylum and genus level. Non-parametric Wilcoxon rank-sum test was used to identify OTUs that shown significant differences in abundance between groups (confidence interval method).

### Accession Number

The 16S rRNA gene sequence information in this paper was deposited in the GenBank Sequence Read Archive database under accession number SRP169602.

## Results

### Characteristics of Sequencing Data

All oropharyngeal samples were analyzed by 16S rRNA gene sequencing, and comparisons were made between healthy piglets and piglets with PRD in the two farms. In this study, 1,491,208 quality-filtered and chimera-checked sequences were obtained with an average length of 446.78 bp across all samples. The mean number of reads per sample was 45,188, ranging from 30,717 to 63,827 reads. A total of 1658 unique OTUs (97% sequence similarity) were detected among all samples. Based on the relative abundance, the taxonomic analysis revealed 22 bacteria phyla, 48 classes, 111 orders, 202 families, and 444 genera across all samples.

All samples’ coverage (Good’s coverage) was more than 99.50%, which indicated that the accuracy of sequencing was reliable. Multiple rarefaction curves were measured using several metrics namely Sobs, Chao1, Simpson, and Shannon, which confirmed adequate sequence coverage for all samples (Supplementary Figure S1). Table 1 indicates the specific bacterial indices (Shannon, Simpson, OTUs, and Chao 1) made from each group as well as the *p*-value for PRD and healthy groups. At the OTU level, there were no statistically significant differences in bacterial diversity and richness between the healthy and PRD groups in the two farms (*p* > 0.05), except for bacterial diversity measured by Shannon index in farm A (*p* < 0.05). The comparison of bacterial indices between all groups is shown in Supplementary Figure S2.

**Table 1:** Bacterial diversity indices (Shannon, Simpson, Chao, and OTUs) are presented as the mean ± standard deviation.

Groups	Shannon	Simpson	OTUs	Chao
PRD_A (*n* = 18)	2.9689 ± 0.4539	0.1192 ± 0.0806	234.94 ± 93.132	329.78 ± 138.77
Healthy_A (*n* = 6)	2.4523 ± 0.2073	0.1702 ± 0.0385	218.83 ± 74.158	318.75 ± 96.737
PRD_B (*n* = 5)	3.3448 ± 0.3253	0.1013 ± 0.0346	373.60 ± 55.810	516.92 ± 99.637
Healthy_B (*n* = 4)	2.9979 ± 0.8019	0.1537 ± 0.1014	530.75 ± 219.99	744.07 ± 266.83
*p*-value (Healthy_A/PRD_A)	0.0141^∗^	0.1531	0.7052	0.8592
*p*-value (Healthy_B/PRD_B)	0.4019	0.3094	0.1625	0.1183


*Student’s *t*-test was performed to evaluate statistical significance. ^∗^*p* < 0.05, ^∗∗^*p* < 0.01, and ^∗∗∗^*p* < 0.001.*

**FIGURE 1:**
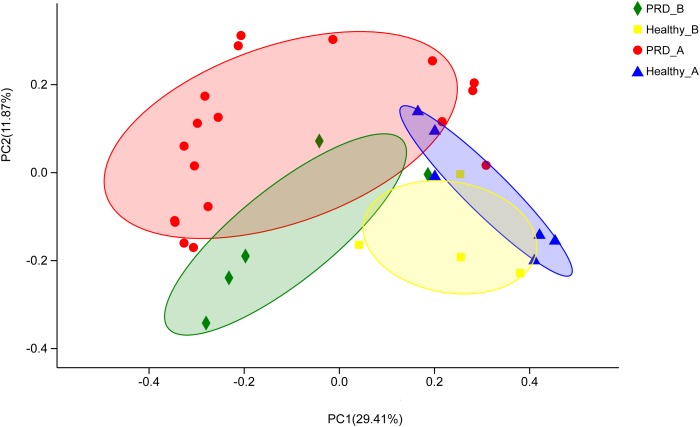
Principal coordinates analysis (PCoA) was performed at the Operational taxonomic unit (OTU) level based on Bray–Curtis metrics for all samples. Each group is represented in a different color and shape.

Beta diversity of each group was calculated through cluster tree analysis and PCoA. The microbial community structure of all samples was analyzed using the phylogeny-based Bray–Curtis method and visualized using PCoA (Figure 1). The first two factors (PC1 and PC2) accounted for 41.28% of the sample variation, which demonstrated that the microbial communities were distinguishable from one another, especially the samples from different farms (Figure 1).

### Characterization of the Oropharyngeal Microbiota Within Different Groups

**FIGURE 2:**
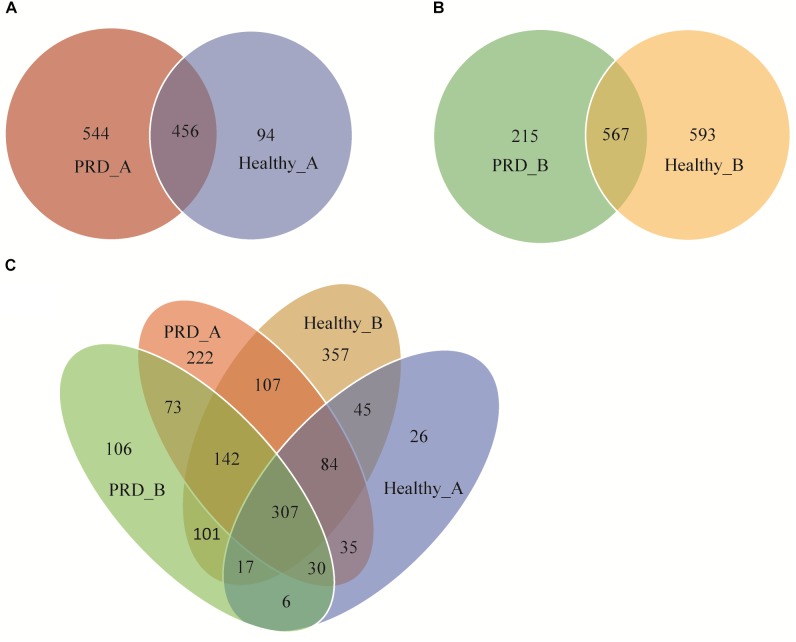
Venn diagrams show the numbers of unique and shared OTUs between Porcine respiratory disease (PRD) and healthy groups in **(A)** farm A, **(B)** farm B, and **(C)** between all four groups.

The Venn diagrams displayed a high number of unique and shared OTUs in suckling piglets. In addition, a clear overlap pattern for oropharyngeal samples from the two farms was also observed (Figure 2). In farm A, 1094 OTUs were identified, of which 456 OTUs (41.68%) were shared between the two groups. The total number of OTUs in the PRD group was much higher than that in the healthy group (1000 OTUs versus 550 OTUs in controls) (Figure 2A). In farm B, 1375 OTUs were identified totally and 567 OTUs (41.24%) were shared between the two groups (Figure 2B). There were 1658 OTUs among all groups, of which 811 OTUs (48.91%) were shared between the two farms regardless of health status (Figure 2C). This illustrated that some common OTUs may be the permanent inhabitants of the oropharynx of piglets.

**FIGURE 3:**
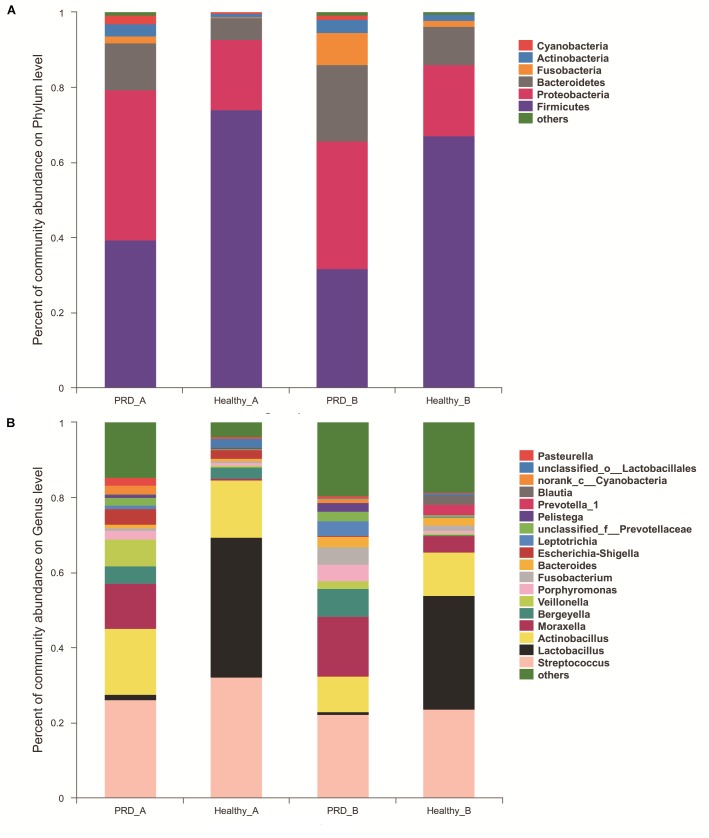
Community bar-plot analysis shows relative abundance of sequences at the **(A)** phylum and **(B)** genus levels observed in PRD and healthy groups in farm A and farm B. OTUs comprising less than 1 and 2% at the phylum and genus level of the total abundance are represented as others.

There were 22 different phyla identified across all samples, of which only five phyla had more than 1% overall relative abundance- *Firmicutes* (53.11%), *Proteobacteria* (27.89%), *Bacteroidetes* (12.17%), *Fusobacteria* (3.15%), and *Actinobacteria* (2.29%), which comprised on average 98.61% of the total reads and formed the main microbiota of porcine oropharynx (Figure 3A). In the Figure 3A, ‘others’ represents bacterial phyla that formed less than 1% of the total abundance. The relative abundance was different at the phylum level in piglets from both farm A and farm B (Supplementary Figure S3). The heatmap profile indicated that oropharyngeal microbial community structures of the four groups were similar at the phylum level (Supplementary Figure S4).

**FIGURE 4:**
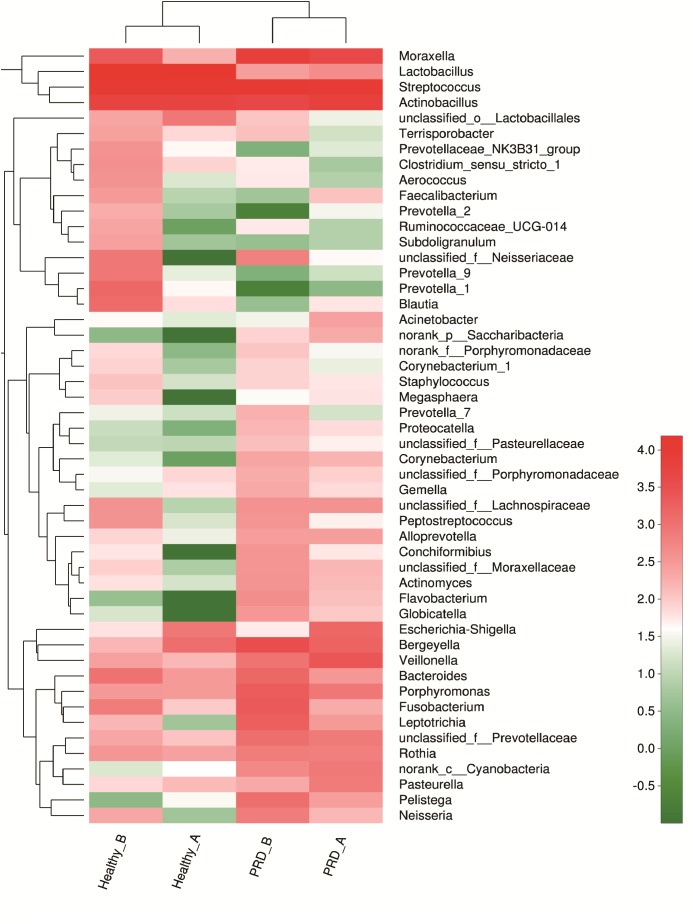
Heatmap analyses of abundant genera in each group (top 50). The bacterial phylogenetic tree was calculated using the neighbor-joining method and the relationship among the four groups was determined by Bray–Curtis distance. The heatmap plot depicts the relative percentage of each bacterial genera (variables clustering on the vertical-axis) within each group (horizon-axis clustering). The color of the spots in the right panel represents the relative values (lg) of the genera in each group.

In farm A, the most abundant bacterial genera in the PRD group were *Streptococcus* (26.25%), *Actinobacillus* (17.63%), *Moraxella* (12.02%), *Veillonella* (7.16%), *Bergeyella* (4.60%), and *Escherichia-Shigella* (4.19%), while *Lactobacillus* (37.28%), *Streptococcus* (32.26%)*, Actinobacillus* (15.09%), *Bergeyella* (3.10%), and *Escherichia-Shigella* (2.24%) were the predominant genera in healthy group (Figure 3B and Supplementary Table S1). In farm B, the prevalent genera found in the PRD group were *Streptococcus* (22.22%), *Moraxella* (15.82%), *Actinobacillus* (9.66%), *Bergeyella* (7.46%), *Fusobacterium* (4.64%), *Porphyromonas* (4.31%), *Leptotrichia* (4.04%), whereas the genera *Lactobacillus* (30.44%), *Streptococcus* (23.60%), *Actinobacillus* (11.48%), *Moraxella* (4.28%), and *Bacteroides* (2.00%) were predominant in the healthy group (Figure 3B and Supplementary Table S1). In the Figure 3B, ‘others’ represents bacterial genera that formed less than 2% of the total abundance. Heatmap analyses of abundant genera in each group are displayed in Figure 4. There was remarkable variability in the oropharyngeal microbial composition at the genus level (Supplementary Figure S5).

There was a relative increase in *Proteobacteria* and *Bacteroidetes* in conjunction to a decrease in *Firmicutes* in the PRD groups from both farms. The increase in *Proteobacteria* corresponded to a higher abundance of *Moraxella* and *Pasteurella* as well as other bacterial components present in low abundance. Meanwhile, the increase in *Bacteroidetes* was mainly due to the relatively higher abundance of *Bergeyella* and *Porphyromonas* in piglets with PRD. In addition, within *Firmicutes*, the principal change was in *Lactobacillus*, which decreased from 37.28 and 30.45% in healthy groups to 1.26 and 0.60% in PRD groups from the two farms, respectively (Supplementary Table S1).

### Core Oropharyngeal Microbiota of Healthy Piglets From the Two Farms

The core microbiota was defined as those OTUs with more than 1% relative abundance and present in all healthy samples. The core microbiota in the oropharynx of healthy piglets from farm A and farm B was described in Table 2. Some remarkable differences were observed in the core genera. *Bergeyella* and *Escherichia-Shigella* were relatively abundant in farm A while *Bacteroides* and *Prevotella_1* were part of the core genera in farm B. The five core oropharyngeal genera for farm A were *Streptococcus*, *Lactobacillus*, *Actinobacillus*, *Bergeyella*, and *Escherichia-Shigella*. For farm B, *Streptococcus*, *Lactobacillus*, *Actinobacillus*, *Bacteroides*, and *Prevotella_1* formed the core oropharyngeal microbiota.

**Table 2:** The core microbiota in the oropharynx of healthy piglets from the two farms.

Farms	Taxonomic level	Phyla	Mean relative abundance (%)
A	Phylum	*Firmicutes*	74.18
		*Proteobacteria*	18.54
		*Bacteroidetes*	5.93
	Genus	*Streptococcus*	32.26
		*Lactobacillus*	37.28
		*Actinobacillus*	15.09
		*Bergeyella*	3.10
		*Escherichia-Shigella*	2.24
B	Phylum	*Firmicutes*	67.07
		*Proteobacteria*	19.00
		*Bacteroidetes*	10.12
	Genus	*Streptococcus*	23.60
		*Lactobacillus*	30.44
		*Actinobacillus*	11.48
		*Bacteroides*	2.01
		*Prevotella_1*	2.72


### Comparison of Oropharyngeal Microbiota Between Healthy and PRD Piglets

To explore the oropharyngeal microbiota differences between the healthy and PRD piglets, non-parametric Wilcoxon rank-sum test was used to compare the mean relative abundance of the predominant bacteria at the phylum and genus levels. Some differences were marked in the microbiota communities between the two groups depending on the health status. In farm A, at the phylum level, there was a greater relative abundance of *Firmicutes* in healthy piglets (38.34% versus 2.45% in PRD group, *p* < 0.05), while in PRD group, we observed a predominance of *Proteobacteria* (40.80% versus 20.20% in healthy group, *p* < 0.05) and *Actinobacteria* (3.15% versus 0.84% in healthy group, *p* < 0.05) (Supplementary Figure S6). In farm B, only *Fusobacteria* was significantly different between the two groups (8.67% in PRD_B versus 1.94% in Healthy_B, *p* < 0.05) (Supplementary Figure S6).

**FIGURE 5:**
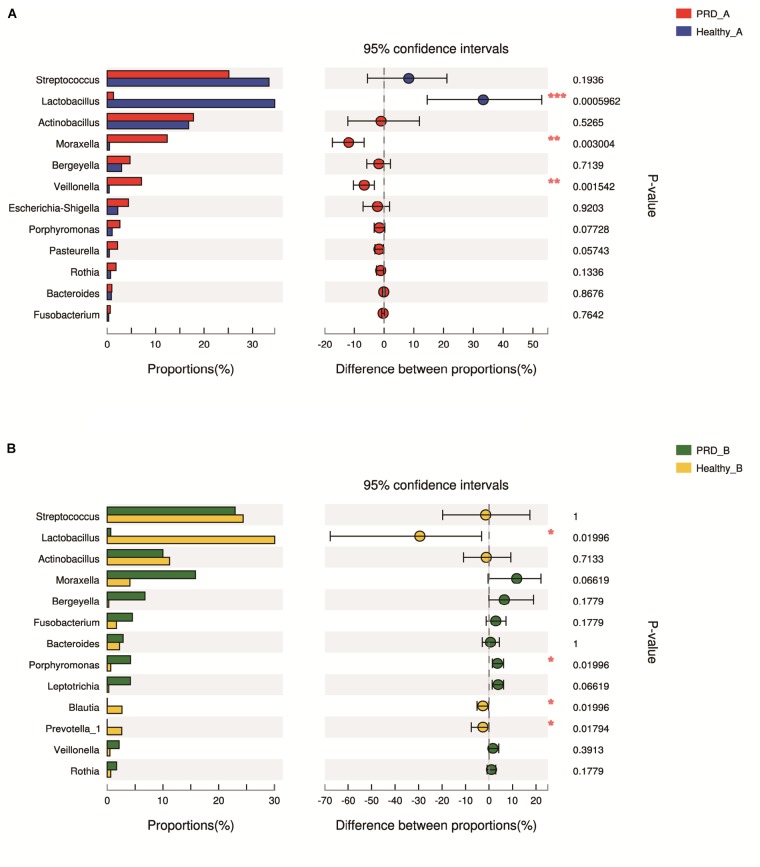
Bar graphs illustrate the differences in predominant bacterial genera between PRD and healthy groups in **(A)** farm A and **(B)** farm B, respectively. Data of PRD and control groups are shown as relative abundance (%) of genera in each group. Statistical analysis was performed by the Wilcoxon rank-sum test. ^∗^*p* < 0.05, ^∗∗^*p* < 0.01, and ^∗∗∗^*p* < 0.001.

In farm A, the percentages of *Moraxella* (12.02%) and *Veillonella* (7.16%) in the PRD group were higher as compared to the healthy group (*p* < 0.05). However, we observed a statistically significant predominance of *Lactobacillus* in the healthy group (37.28% versus 1.26% in PRD group, *p* < 0.05) (Figure 5A and Supplementary Table S1). In farm B, the genus *Porphyromonas* (4.31% versus 0.62% in Healthy_B, *p* < 0.05) was predominant in PRD group, whereas *Lactobacillus* (30.45% in Healthy_B versus 0.60% in PRD_B) was statistically increased in healthy group as well as *Blautia* and *Prevotella_1* (*p* < 0.05) (Figure 5B and Supplementary Table S1). Therefore, the data demonstrated that the oropharyngeal microbial community composition differed significantly between the PRD and healthy piglets and between different farms.

## Discussion

Most microbiota studies on pigs have focused on the intestinal tract, while only a few studies exist on the microbiota of the upper respiratory tract. In this study, we used 16S rRNA sequencing to generate an overview of the oropharyngeal microbiota of suckling piglets and to study the effect of respiratory diseases on this population.

We observed increased alpha diversity in the bacterial population of the oropharynx of piglets with PRD in the two farms compared to the controls. In human patients with cirrhosis and pneumonia, the bacteria diversity increased significantly (Lu et al., 2015). It is possibly because the pathogenic bacteria lead to a modification and an enrichment of oropharyngeal microbiota. In addition, beta-diversity analysis revealed that the oropharyngeal microbial communities in PRD groups were distinguishable from those in healthy groups in phylogenetic space as judged by Bray–Curtis distance, suggesting that disease conditions may be one of the important factors accounting for the change in microbial structure. In addition, it was reported that farm management (diet and antimicrobial use) could influence the nasal microbiota of pigs (Weese et al., 2014). We also found that a very high number of OTUs were shared between the two farms. The existence of shared bacteria are evidence for the importance of the regulation of upper airways’ microbiota by the host.

Since the oropharynx is contiguous with the oral cavity and nasopharynx, it is continuously exposed to bacteria from the saliva, inhaled air, ingested food, and airway surface liquids of the respiratory tract (Humphreys and McBain, 2013). The analysis of core microbiota is performed to find colonizers of the oropharynx of piglets. From our results, the genera *Streptococcus*, *Lactobacillus*, and *Actinobacillus* comprised a relatively high abundance of total sequences and were the common bacteria in the two farms, which could be the permanent inhabitants of the porcine oropharynx. *Streptococcus* *suis*, *Haemophilus parasuis*, and *Actinobacillus suis* are early colonizers of the respiratory tract in humans and they are often isolated from the upper respiratory tract of healthy pigs (Brockmeier et al., 2017). In previous studies, *Streptococcus*, *Actinobacillus*, *Pasteurella*, and *Veillonella* formed the core microbiome of tonsils and *Lactobacillus* presented more than 1% abundance in nasal samples from piglets (Lowe et al., 2012; Slifierz et al., 2015). These bacteria were also the predominant genera in our study, reflecting the microbiota similarities of the porcine upper respiratory tract. In healthy individuals, the core oropharyngeal microbiome contained *Streptococcus*, *Prevotella*, *Neisseria*, *Actinobacillus*, *Rothia*, and *Veillonella* (Andersson et al., 2008; Hang et al., 2017), suggesting that the oropharyngeal microbiota is relatively similar in humans and piglets. These core genera well adapted to the oropharynx may serve as potential markers of a typical swine oropharyngeal microbiota.

In addition to characterizing the core microbiota, we compared the differences in the oropharyngeal microbiota between healthy and PRD piglets. In farm A, the relative abundances of *Moraxella* and *Veillonella* were higher in piglets with PRD, while only *Porphyromonas* was relatively abundant in the PRD group from farm B. Similar to our results, the microbiota in asthma patients shows signs of dysbiosis compared to that in healthy subjects with an increase in *Proteobacteria*, particularly *Moraxella* (Depner et al., 2017). The higher relative abundances of *Moraxella*, *Veillonella*, and *Porphyromonas* in PRD groups suggest that these taxa along with other pathogens may be associated with the development of respiratory diseases.

In the two farms, the genus *Moraxella* was prevalent in the PRD groups. *Moraxella* is recognized as an important pathogen of respiratory tract infections in humans, including childhood asthma (Depner et al., 2017). Interestingly, a novel species, *Moraxella porci*, was isolated from different clinical specimens in diseased porcine tissues, including lungs and brain (Vela et al., 2010). The results of this study suggest that *Moraxella* deserves considerable attention as a potential opportunistic pathogen in the porcine respiratory tract and further research should focus on it.

The relative abundance of *Porphyromonas* was increased in the PRD groups from both farms. *Porphyromonas gingivali*s has been identified as a main microbial factor for causation and progression of periodontal disease (Morozumi et al., 2016). The cell wall components of *P. gingivalis*, especially lipopolysaccharide, can activate host immune responses, including the production of pro-inflammatory cytokines, anti-inflammatory cytokines, and chemokines (Sun et al., 2010). Meanwhile, the relative abundance of *Veillonella* was also increased in the PRD groups. *Veillonella* species have generally been regarded as commensal bacteria in the gastrointestinal, genitourinary, and respiratory tracts of humans and animals (Chen et al., 2016). Several studies have linked them to oral diseases, even though the pathogenic roles of *Veillonella* in oral infections have not yet been fully clarified (Kanasi et al., 2010; Mashima et al., 2011). In addition, *Veillonella* species are associated with severe acute and chronic infections, such as meningitis, prosthetic joint infections, and pleuropulmonary infections (Pustelny et al., 2014). The two bacterial genera may be considered as the bacterial markers of the health status and the potential roles of the two bacteria in PRD warrant further investigation.

Another genus, *Pasteurella*, was relatively abundant in the diseased groups from the two farms, even though no statistical significance was observed (*p* > 0.05). *P. multocida* is an opportunistic pathogen and a common inhabitant of the upper respiratory tract of many animals, and a causative agent of numerous important diseases, including respiratory diseases (Hansen et al., 2010; Liu et al., 2017). In addition, a high prevalence of *P. multocid*a was demonstrated from cases of porcine bronchopneumonia, which was considered a secondary pathogen, dependent on co-infections or immunosuppression of the host (Pors et al., 2011). In our study, *Pasteurella*, together with other genera, may be responsible for the respiratory diseases.

Notably, we observed a statistically significant predominance of *Lactobacillus* in healthy groups from the two farms (*p* < 0.05). *Lactobacillus* are commonly investigated as probiotic agents, which can increase natural killer cell activity, reduce proinflammatory cytokine production, produce bacteriocins, and protect biological niches (Goldstein et al., 2015; Burrough et al., 2017; Niederwerder, 2017). In addition, dietary supplementation with *lactobacilli* has been shown to enhance immune response and protect against respiratory tract pathogen challenge (Mortaz et al., 2013). Similar to our study, *Lactobacillus* was more abundant in inoculated pigs that did not develop dysentery when compared with diseased pigs (Burrough et al., 2017). Whether the genus *Lactobacillus* is protective against PRD and may be used as a respiratory probiotic, through effects on pathogen inhibition or host immunomodulation, requires further evaluation. *Lactobacillus* supplementation in the feed may be a potential strategy for generating probiotic-mediated protection against PRD.

Although similar disease-related shifts in microbiota composition were observed from the two farms, it is difficult to conclude from our results whether the presence of an altered microbiome is the cause or a result of the respiratory diseases. Investigation into the potential role of the observed species in relation to increased or decreased risk of developing respiratory diseases and thereby the utility of their detection in the microbiota in evaluating potential prebiotic approaches to PRD control is warranted.

## Conclusion

In conclusion, this study analyzed the oropharyngeal microbiota of healthy suckling piglets and piglets with respiratory diseases for the first time. Our results revealed a core oropharyngeal microbiome in healthy piglets, including *Streptococcus*, *Lactobacillus*, and *Actinobacillus*. In addition, we suggest that the genera *Moraxella*, *Veillonella*, and *Porphyromonas* may play a potential role in PRD. The statistically significant predominance of *Lactobacillus* in healthy piglets implies a potential protective role against PRD and administration of probiotics to maintain stability in the oropharyngeal microbiota might be a useful strategy to prevent or treat respiratory diseases.

## Author Contributions

HY and QW conceived the study and wrote the paper. AH, XW, WQ, and QW performed the experiments. AH, XW, HY, and QW analyzed the sequence data. LS, RC and CL contributed to reagents and materials. HY, LS and CL revised this manuscript critically for important content. All authors have read and approved the content of the manuscript.

## Conflict of Interest Statement

The authors declare that the research was conducted in the absence of any commercial or financial relationships that could be construed as a potential conflict of interest.

## Footnotes

1 http://drive5.com/uparse/

2 http://rdp.cme.msu.edu/

3 www.mothur.org/
