# Supplementary material for: Comparison of Oropharyngeal Microbiota in Healthy Piglets and Piglets With Respiratory Disease
Source: Front Microbiol. 2018 Dec 21;9:3218. doi: 10.3389/fmicb.2018.03218 (PMC6309737; doi:10.3389/fmicb.2018.03218)
Supplement: Supplementary file 2 [file Table_2.docx]

***Supplementary Material***

**Comparison of oropharyngeal microbiota in healthy piglets and piglets with respiratory disease**

Qun Wang^1*^, Rujian Cai^2*^, Anni Huang^1^, Xiaoru Wang^1^, Wan Qu^1^, Lei Shi^3, 4^, Chunling Li^2**^, He Yan^1,4**^

*^1^ College of Food Science and Engineering, South China University of Technology, Guangzhou 510641, China*

*^2^ Institute of Animal Health Guangdong Academy of Agricultural Sciences, Guangzhou 510640, Guangdong, China*

*^3^* *Institute of Food Safety and Nutrition, Jinan University, Guangzhou 510632, China*

*^4^ State key Laboratory of Food Safely Technology for Meat Products, Xiamen 361000, Fujian, China*

*These authors contributed equally

**Corresponding author:

Dr. Chunling Li, Ph.D.

Mailing address: Institute of Animal Health Guangdong Academy of Agricultural Sciences, Guangzhou 510640, Guangdong, China

E-mail Address: lclclare@163.com (Chunling Li)

Tel: +86-20-85291377

Dr. He Yan, Ph.D.

Mailing address: School of Food Science and Engineering, South China University of Technology, Guangzhou 510640, P.R. China

E-mail Address: yanhe@scut.edu.cn (He Yan)

Tel: +86-20-87113848; fax: +86-20-8711273

**Supplemental Figures**

**
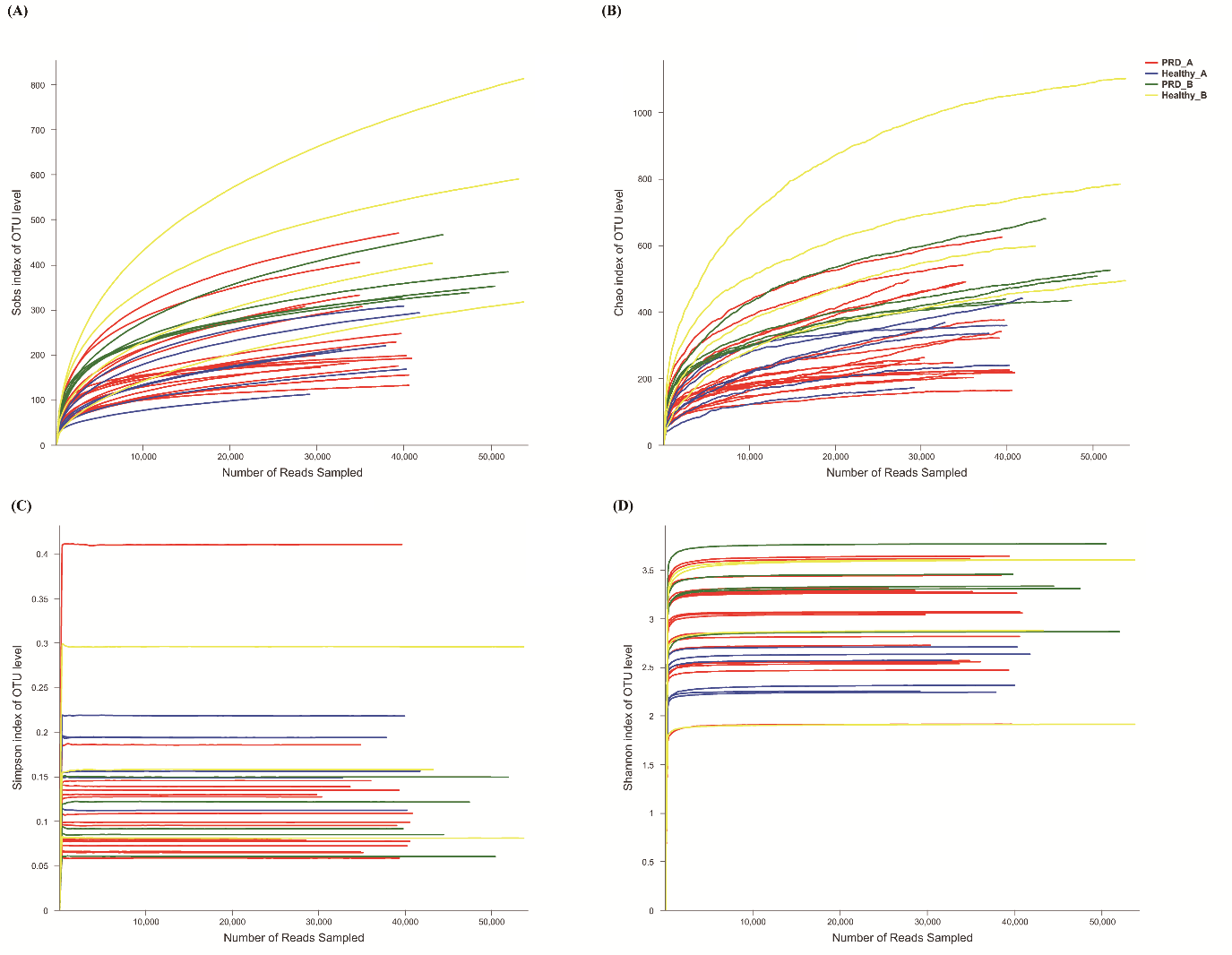
Supplementary Figure 1.** Multiple rarefaction curves of sequences obtained from all samples for (A) Sobs, (B) Chao 1, (C) Simpson, and (D) Shannon index.


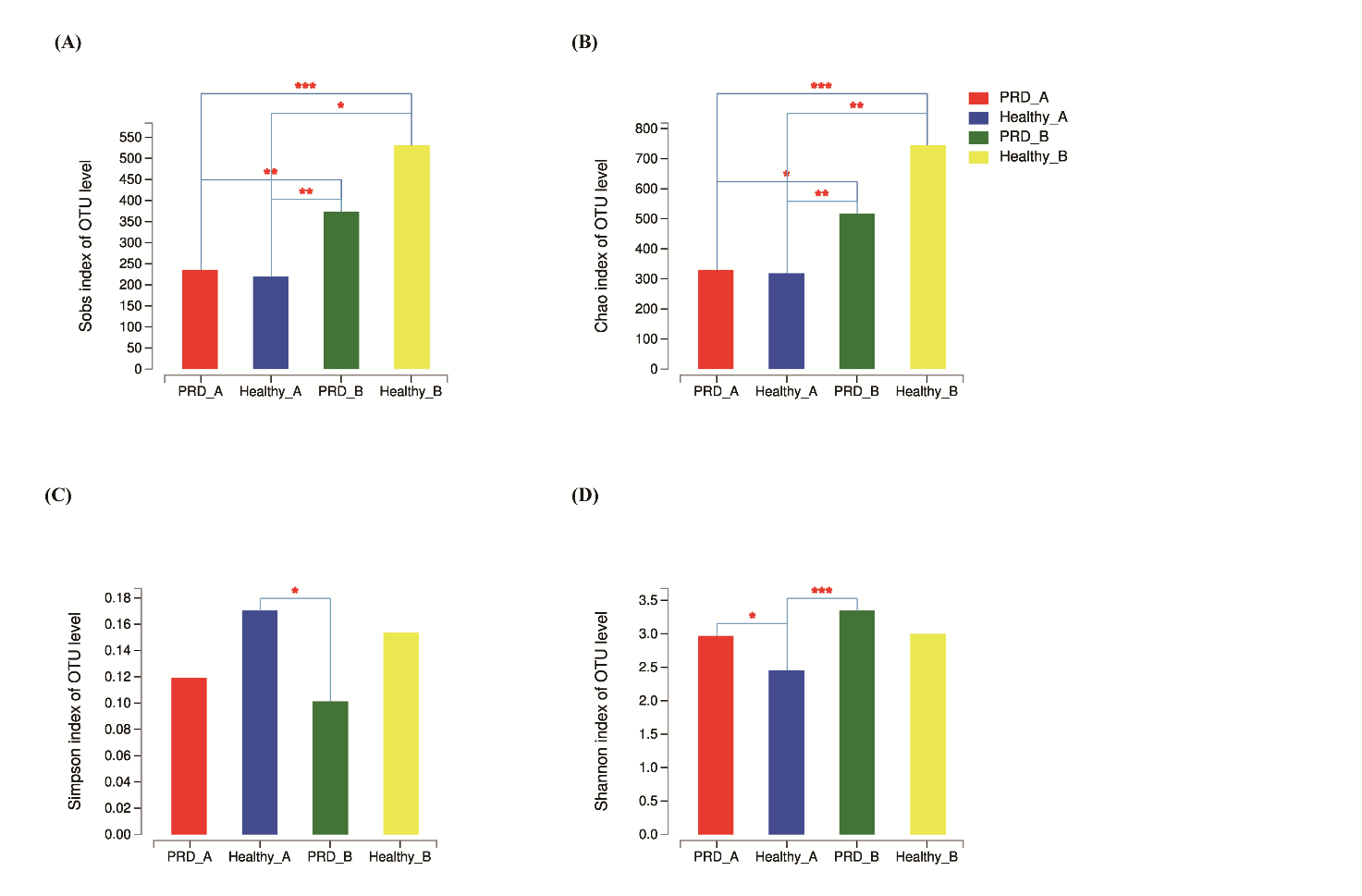


**Supplementary Figure 2.** Bar graphs show the relative abundance measured by (A) Sobs, (B) Chao 1, (C) Simpson, and (D) Shannon index for four groups using Student’ t-test. * *p* < 0.05, ***p* < 0.01, *** *p* < 0.001.


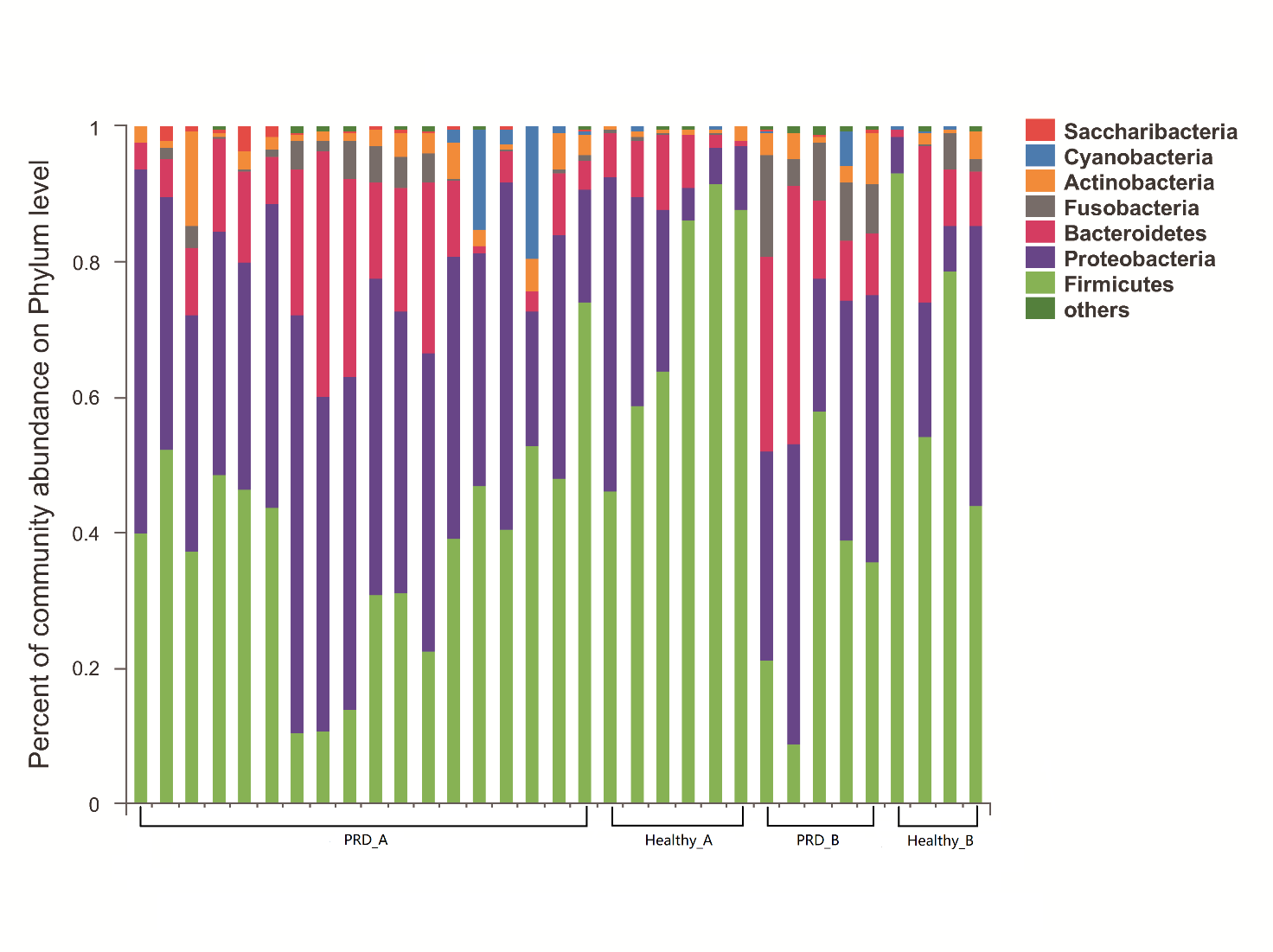


**Supplementary Figure 3.** Relative abundance of sequences at the phylum level observed in all oropharyngeal samples from piglets that developed PRD and healthy piglets from the two farms. Bacterial phyla comprising less than 1% of the total abundance are represented as others.

**Supplementary Figure 4.** Heatmap analyses of abundant phyla in each group. The bacterial phylogenetic tree was calculated using the neighbor-joining method and the relationship among the four groups was determined by Bray-Curtis distance. The heatmap plot depicts the relative percentage of each bacterial phyla (variables clustering on the vertical-axis) within each group (horizon-axis clustering). The color of the spots in the right panel represents the relative values (lg) of the phyla in each group.


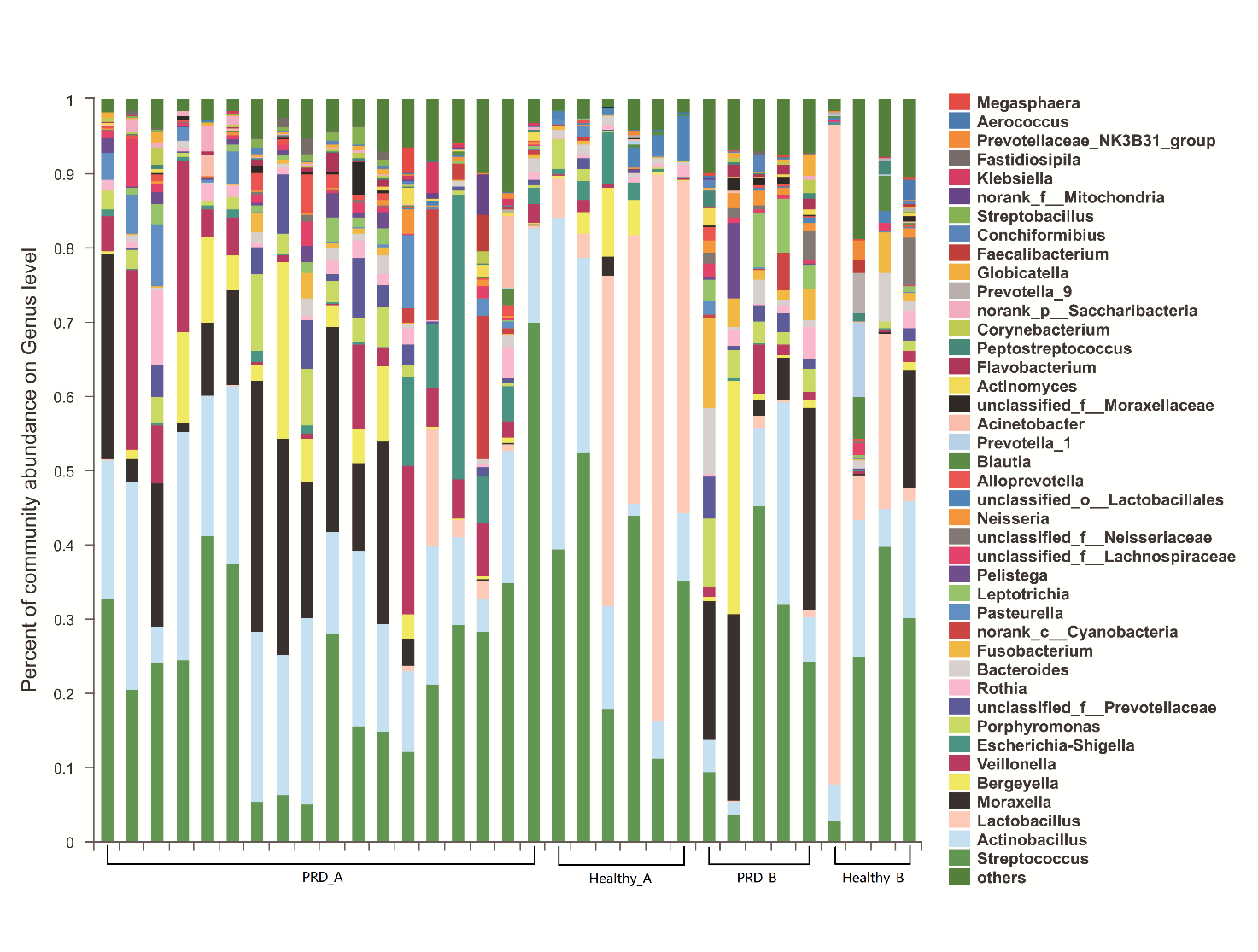


**Supplementary Figure 5.** Relative abundance of sequences at genus level observed in all oropharyngeal samples from piglets that developed PRD and healthy piglets from the two farms. Bacterial genera comprising less than 2% of the total abundance are represented as others.

(A)


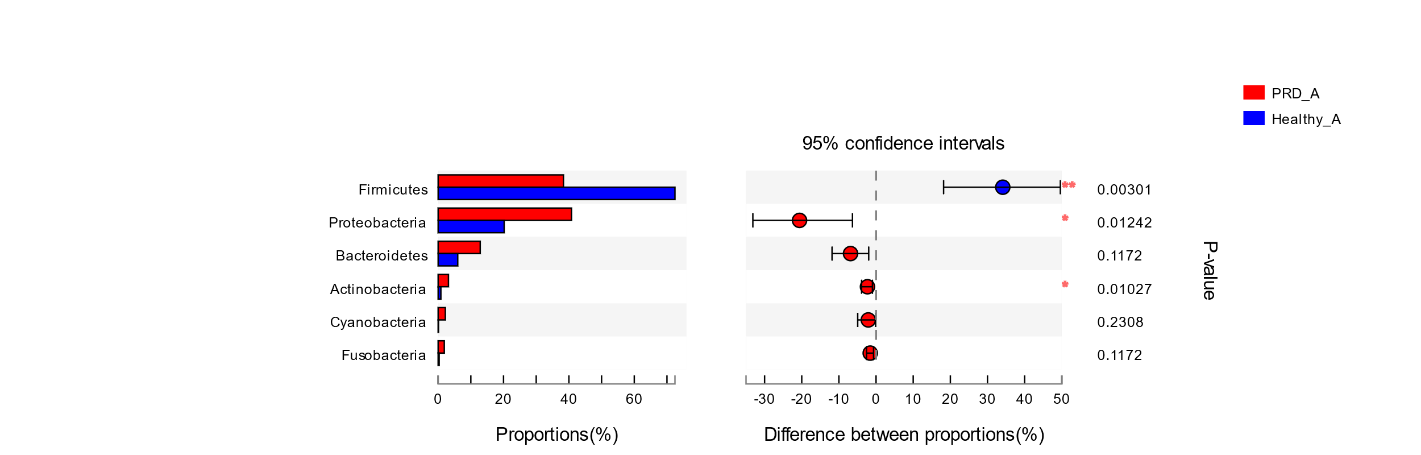


(B)


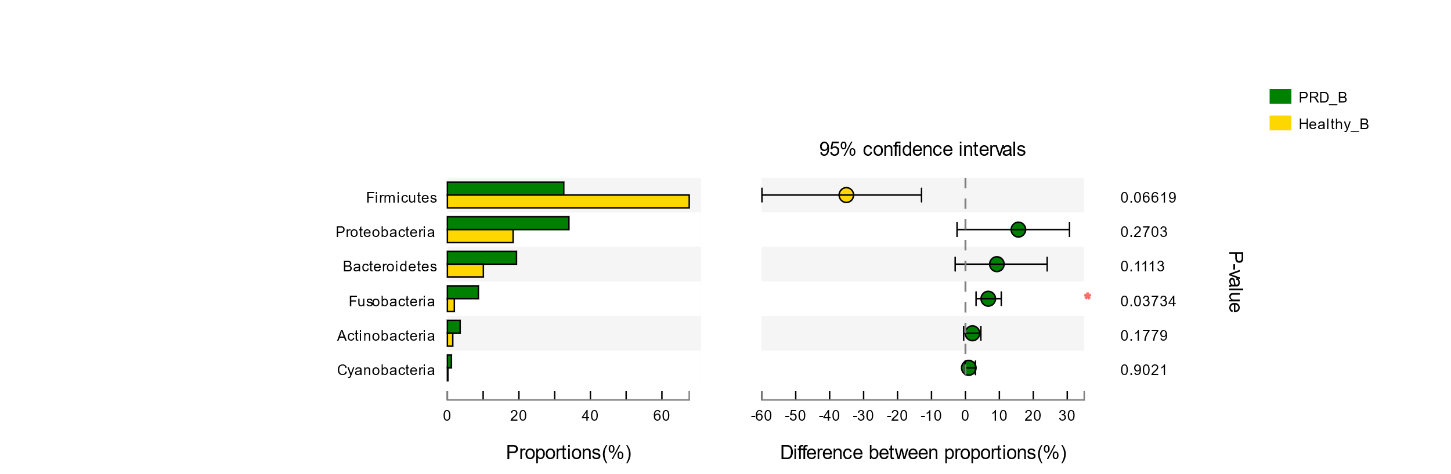


**Supplementary Figure 6.** Bar graphs illustrate the differences in predominant bacterial phyla between PRD and healthy groups in farm A and farm B, respectively. Data of PRD and control groups are shown as relative abundance (%) of phyla in each group. Statistical analysis was performed by the Wilcoxon rank-sum test. * *p* < 0.05, ** *p* < 0.01, *** *p* < 0.001.

**Supplementary Table 1.** Relative abundance of the most common OTUs from the four groups in the two farms. Statistical analysis was performed by the Wilcoxon rank-sum test. **p* < 0.05, ** *p* < 0.01, *** *p* < 0.001.

|  |  | Relative abundance（%） | | | | | |
| --- | --- | --- | --- | --- | --- | --- | --- |
| Phylum | Genus | PRD-A | Healthy-A | *p*-value | PRD-B | Healthy-B | *p*-value |
| *Firmicutes* | *Streptococcus* | 26.25 | 32.26 | 0.1936 | 22.22 | 23.60 | 1.0000 |
|  | *Lactobacillus* | 1.26 | 37.28 | 0.0006*** | 0.60 | 30.45 | 0.0199* |
|  | *Veillonella* | 7.16 | 0.39 | 0.0015** | 2.10 | 0.49 | 0.3913 |
|  | *Blautia* | 0.16 | 0.18 | 0.3501 | 0.009 | 2.54 | 0.0199* |
| *Proteobacteria* | *Actinobacillus* | 17.63 | 15.09 | 0.5265 | 9.66 | 11.48 | 0.7133 |
|  | *Moraxella* | 12.02 | 0.48 | 0.0030** | 15.82 | 4.28 | 0.0662 |
|  | *Escherichia-Shigella* | 4.19 | 2.24 | 0.9203 | 0.11 | 0.12 | 0.5403 |
|  | *Pelistega* | 0.78 | 0.09 | 0.0900 | 2.41 | 0.007 | 0.0179* |
|  | *Pasteurella* | 2.13 | 0.38 | 0.0574 | 0.44 | 0.15 | 0.7133 |
|  | *Neisseria* | 0.44 | 0.01 | 0.0008*** | 1.52 | 0.42 | 0.0662 |
| *Bacteroidetes* | *Bergeyella* | 4.60 | 3.10 | 0.7139 | 7.46 | 0.30 | 0.1779 |
|  | *Porphyromonas* | 2.44 | 0.85 | 0.0773 | 4.31 | 0.62 | 0.0199* |
|  | *Bacteroides* | 0.92 | 0.82 | 0.8676 | 2.96 | 2.01 | 1.0000 |
|  | *Prevotella_1* | 0.01 | 0.11 | 0.0172* | 0.0008 | 2.72 | 0.0179* |
| *Fusobacteria* | *Fusobacterium* | 0.54 | 0.26 | 0.7642 | 4.64 | 1.46 | 0.1779 |
|  | *Leptotrichia* | 0.86 | 0.01 | 0.0084** | 4.04 | 0.30 | 0.0662 |
| *Actinobacteria* | *Rothia* | 1.82 | 0.68 | 0.1336 | 1.66 | 0.63 | 0.1779 |
