# Supplementary figures and images for: Comparison of Oropharyngeal Microbiota in Healthy Piglets and Piglets With Respiratory Disease
Source: Front Microbiol. 2018 Dec 21;9:3218. doi: 10.3389/fmicb.2018.03218 (PMC6309737; doi:10.3389/fmicb.2018.03218)

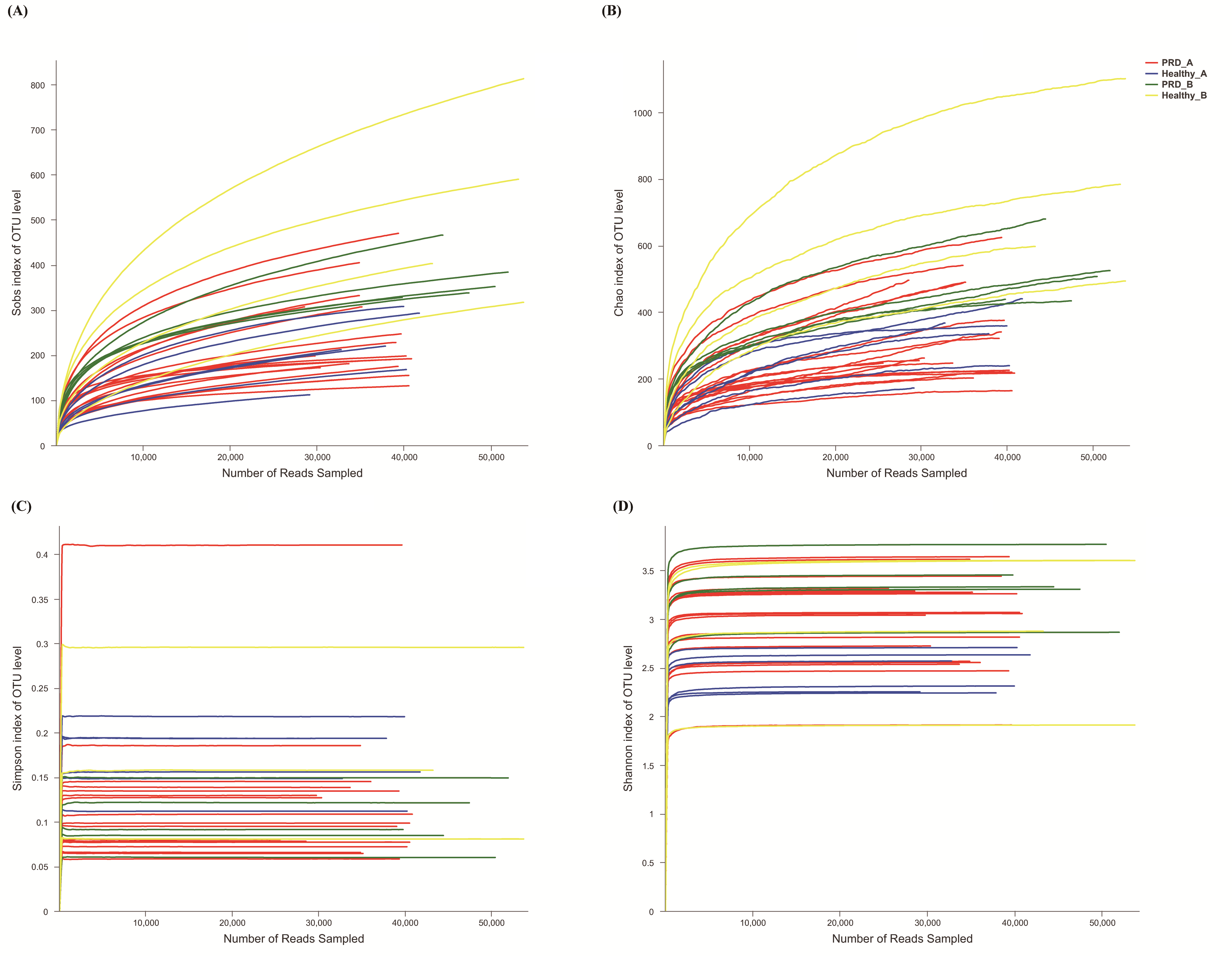

Supplement: Supplementary file 4 [file Image_1.tif]

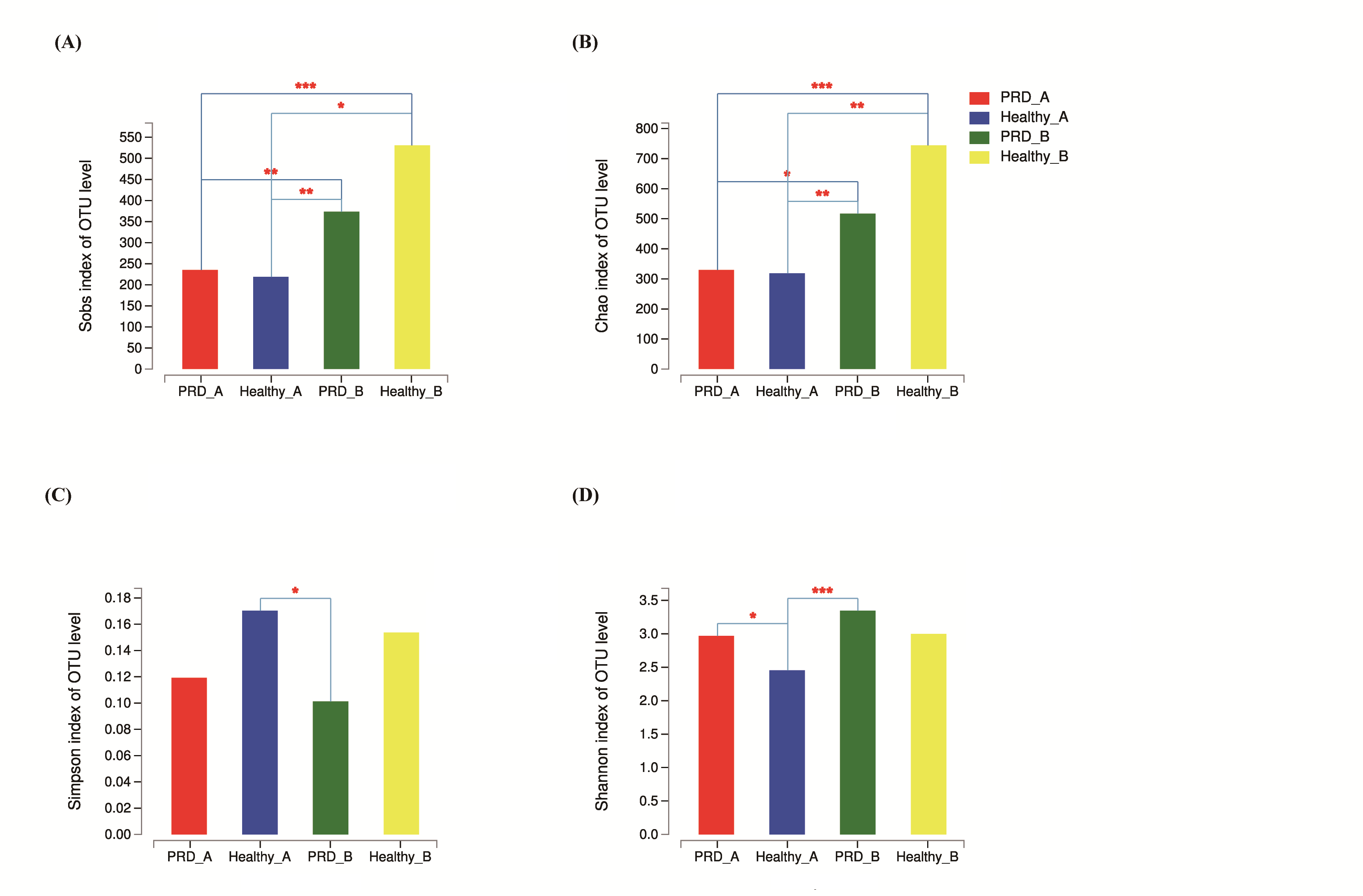

Supplement: Supplementary file 5 [file Image_2.tif]

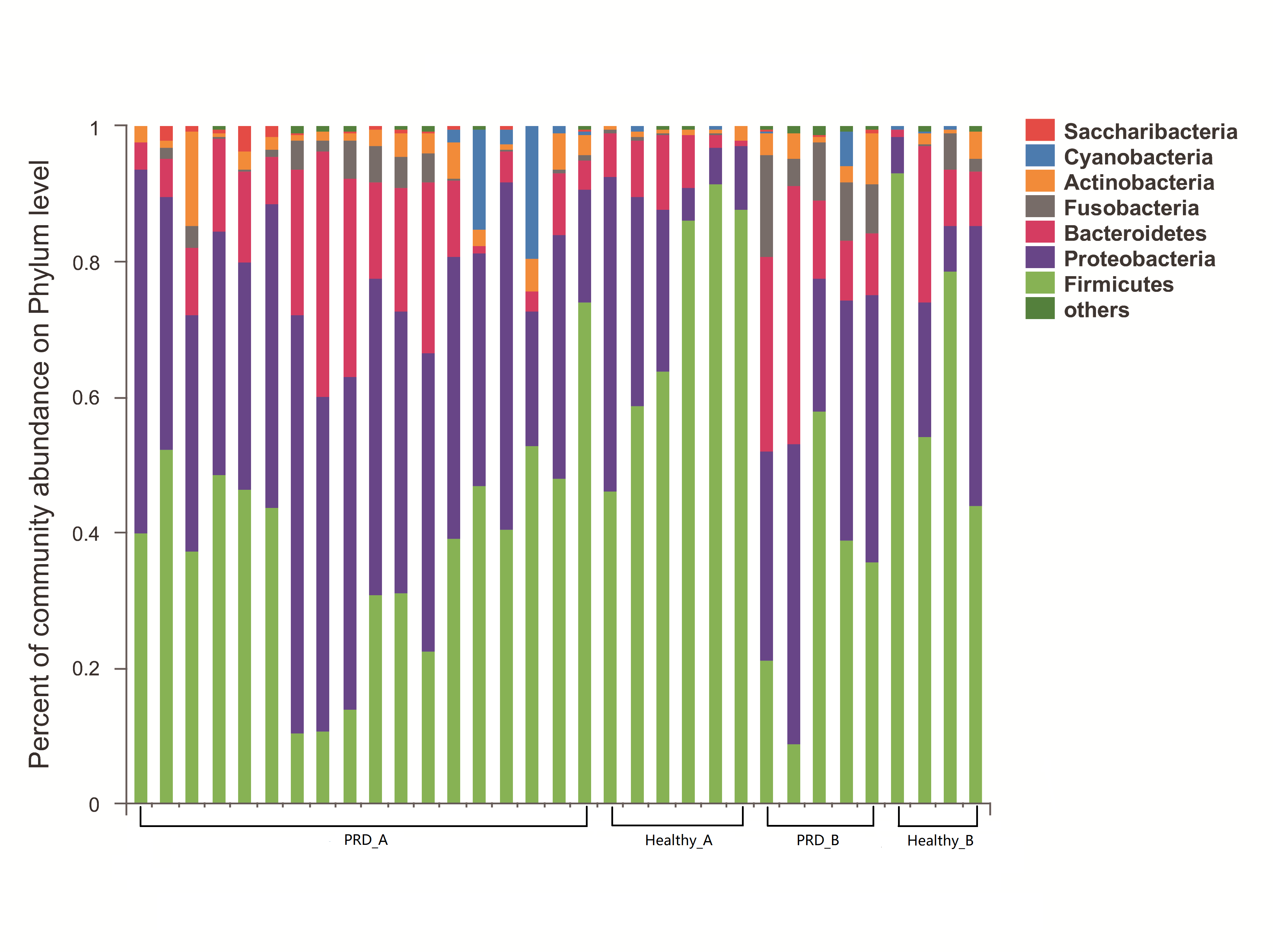

Supplement: Supplementary file 6 [file Image_3.tif]

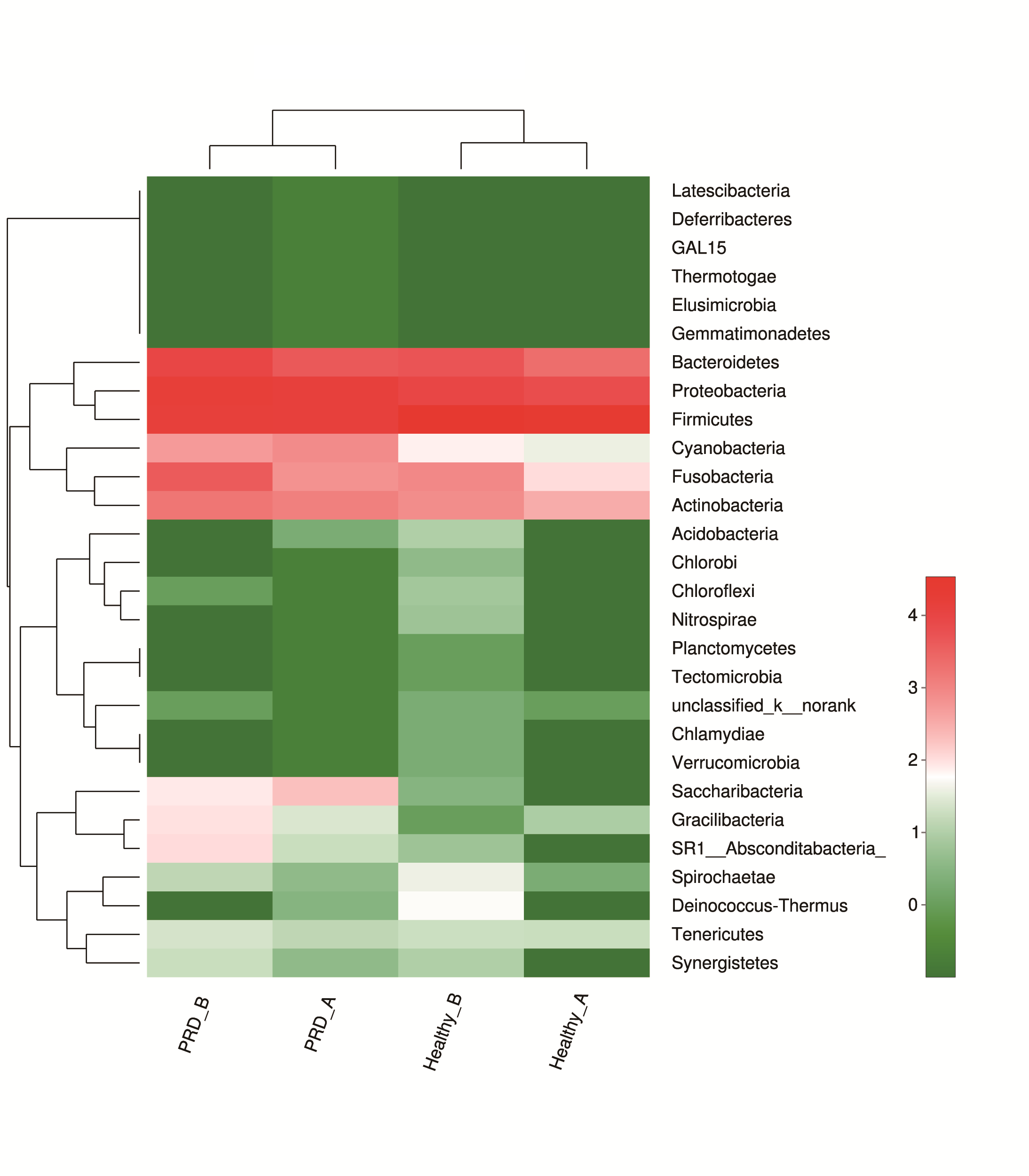

Supplement: Supplementary file 7 [file Image_4.tif]

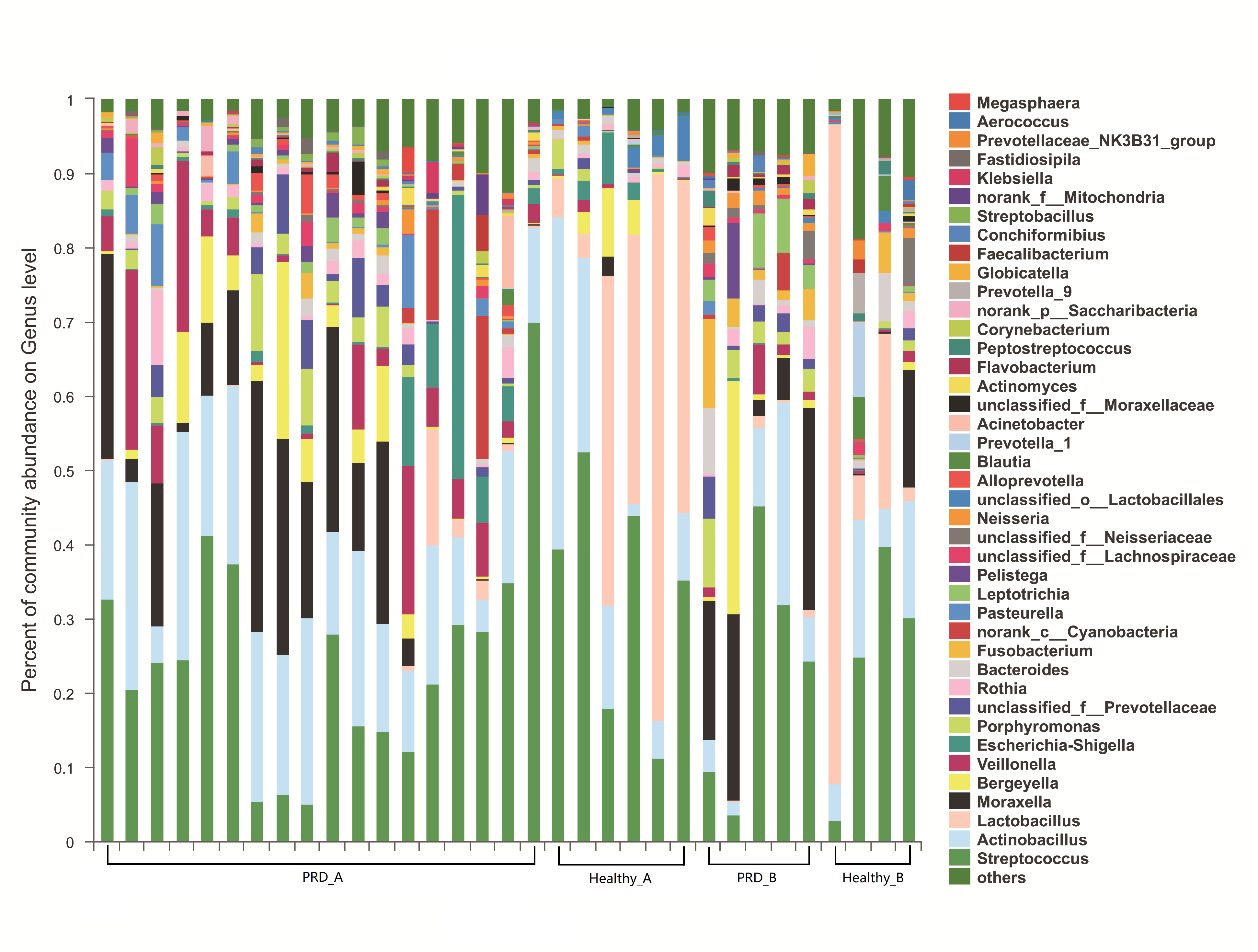

Supplement: Supplementary file 8 [file Image_5.tif]

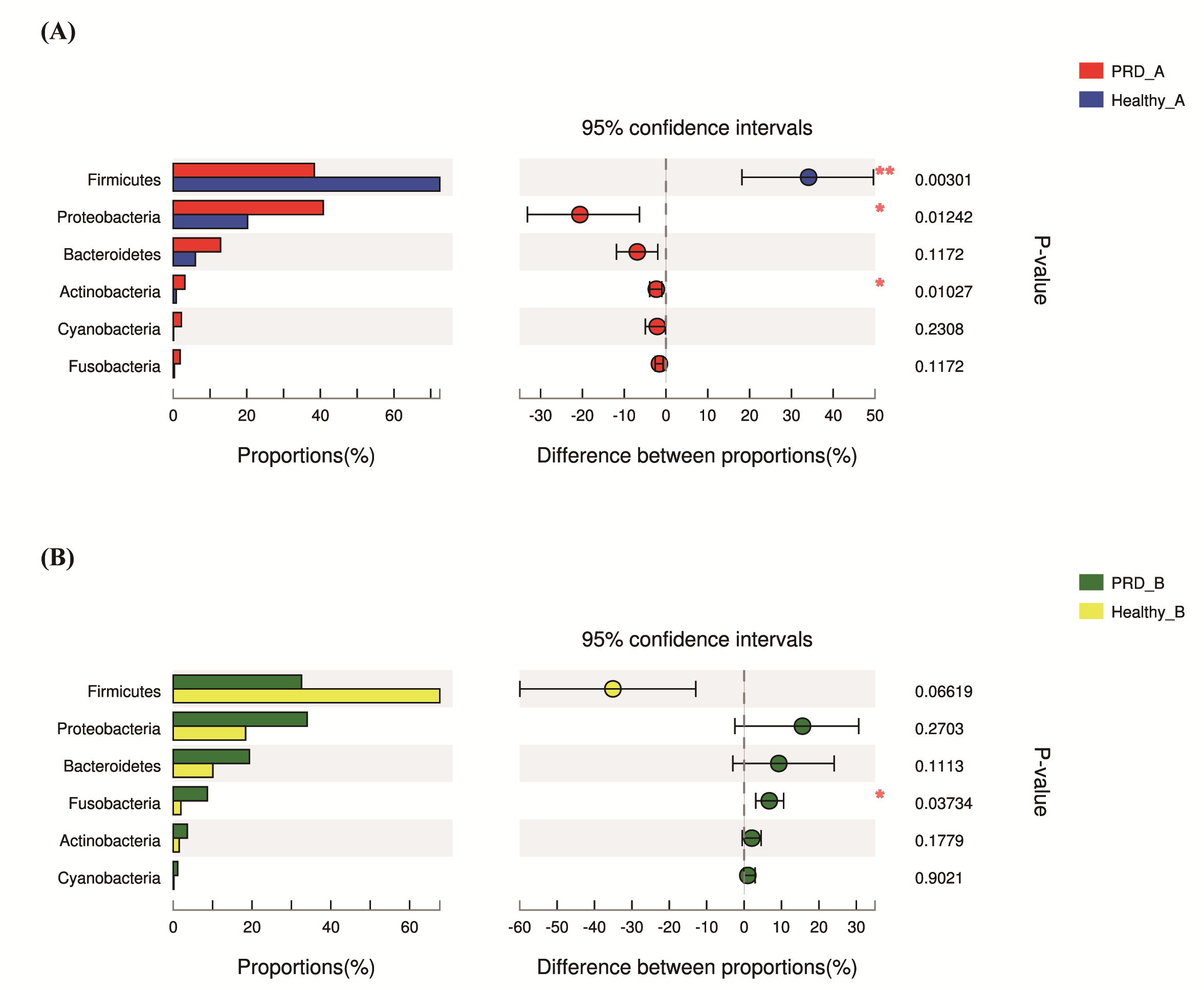

Supplement: Supplementary file 9 [file Image_6.tif]
